# Polyphenols Isolated from Propolis Augment TRAIL-Induced Apoptosis in Cancer Cells

**DOI:** 10.1155/2013/731940

**Published:** 2013-03-19

**Authors:** Ewelina Szliszka, Wojciech Krol

**Affiliations:** Department of Microbiology and Immunology, Medical University of Silesia in Katowice, Jordana 19, 41 808 Zabrze, Poland

## Abstract

Epidemiological data support the concept that phenols and polyphenols in diet are safe and nontoxic, and have long-lasting beneficial effects on human health. The potential target for complementary and alternative medicine (CAM) research has been on the discovery of natural compounds that can be used in the prevention and treatment of cancer. Propolis is one of the richest sources of plant phenolics (flavonoids and phenolic acids). The ethanolic extract of propolis (EEP) and its polyphenols possess immunomodulatory, chemopreventive, and antitumor effects. Tumor necrosis factor-related apoptosis inducing ligand (TRAIL) is a  naturally occurring anticancer agent that preferentially induces apoptosis in cancer cells and is not toxic to normal cells. Endogenous TRAIL plays a significant role in immunosurveillance and defense against cancer cells. However, as more tumor cells are reported to be resistant to TRAIL-mediated death, it is important to develop new strategies to overcome this resistance. EEP and polyphenols isolated from propolis have been shown to sensitize cancer cells to TRAIL-induced apoptosis. In this paper we demonstrate for the first time the crucial role of the main phenolics isolated from propolis in enhancing TRAIL-mediated death in tumor cells for cancer chemoprevention.

## 1. Introduction

The induction of cancer cell-specific apoptosis *via* the activation of TRAIL (tumor necrosis factor-related apoptosis-inducing ligand) signaling has become an important focus of cancer research [1, 2]. However, as more tumor cells are reported to be resistant to TRAIL-mediated death, it is necessary to develop new strategies to overcome this resistance [3–6]. Propolis and its phenolic components exert anticancer and chemopreventive properties by multiple mechanism of action affecting apoptotic pathways in cancer cells [7, 8]. Extracts of propolis and polyphenols isolated from propolis have been shown to sensitize cancer cells to TRAIL-induced apoptosis [9–11]. In this paper, we summarize the evidence for the crucial role of the main phenolics isolated from propolis in enhancing TRAIL-mediated death in tumor cells for cancer chemoprevention.

## 2. Propolis and Its Polyphenolic Constituents as Cancer Chemopreventive Agents

Propolis (bee glue) is a resinous hive product collected by honey bees from many plant sources. The chemical composition of propolis is complex and largely depends on the geographical origin and specific flora at the site of collection [12]. It usually contains a variety of different compounds, including phenolic acids or their esters, flavonoids (flavones, flavanones, isoflavones, flavonols, dihydroflavonols, chalcones), terpenes, aromatic aldehydes and alcohols, fatty acids, stilbenes, and *β*-steroids [13, 14].

Propolis is one of the richest sources of plant phenolics (flavonoids and phenolic acids) [9, 13]. Cinnamic acid, *o*-coumaric acid, *m*-coumaric acid, *p*-coumaric acid, ferulic acid, isoferulic acid, caffeic acid, caffeic acid phenylethyl ester (CAPE), chrysin, tectochrysin, apigenin, acacetin, naringenin, rhamnetin, pinocembrin, pinostrobin (pinocembrin-7-methylether), pinobanksin, sakuranetin, isosakuranetin, galangin, kaempferol, kaempferide, quercetin have been reported to be identified in European propolis (Croatian, Dutch, Polish, Portuguese, and Slovenian) [8–10, 15–19]. The main constituents of Brazilian green propolis are *p*-coumaric acid, ferulic acid, cinnamic acid, and its derivative—drupanin, baccharin, and artepillin C, chrysin, tectochrysin, pinocembrin, pinobanksin, isosakuranetin, kaempferol, kaempferide, and quercetin [20–23]. Brazilian red propolis is abundant with pinocembrin, pinobanksin, liquiritigenin, naringenin, daidzein, formonetin, biochanin A, quercetin, rutin, and isoliquiritigenin [24]. Chinese propolis contains CAPE, chrysin, and pinocembrin at high concentration [25].

Propolis as a harmless natural product has been used in folk medicine since ancient times and recently, it became a subject of special interest in the area of oncological research as a source of valuable polyphenolic compounds for the prevention and treatment of cancer [7, 8, 26]. Propolis cannot be used as raw material and it must be purified by extraction to remove the inert material and preserve the polyphenolic fraction [10]. The ethanolic extract of propolis (EEP) and its phenolics possess immunomodulatory, anticancer, and chemopreventive properties [27–35].

Chemoprevention is a means of cancer control in which carcinogenesis is inhibited or reversed by nutritional or pharmacological intervention with natural or synthetic agents [36–38]. When Dr. Michael Sporn for the first time introduced the term “chemoprevention,” referring to the activity of natural forms of vitamin A and its synthetic analogs in preventing the development and progression of epithelial cancer, he originated a novel field in cancer research [39]. 

Several mechanisms contribute to the overall cancer preventive and antitumor properties of propolis. EEP and its phenolic components suppress proliferation and tumor growth, induce cell-cycle arrest and apoptosis in cancer cells [7–11, 26–29]. The role of propolis in host immune functions against tumor onset has become increasingly recognized in our understanding of the mechanisms of cancer prevention. EEP stimulates nonspecific immunity, activates humoral immunity, and enhances cell-mediated immunity [30]. In our opinion the immunomodulatory effect of propolis and its phenolic components is also evoked by targeting of TRAIL-induced apoptosis in cancer cells for cancer chemoprevention. We have shown that TRAIL-resistant cancer cells can be sensitized by EEP and its phenolic components [9–11]. 

## 3. Characteristics of TRAIL and Death Receptors 

The death ligand TRAIL (tumor necrosis factor-related apoptosis-inducing ligand), a member of the TNF superfamily, induces apoptosis in cancer cells with no toxicity against normal tissues [40–43]. TRAIL was discovered independently by two teams, both of which reported sequence homology with the extracellular domain of the other TNF family members: FasL (CD95L/Apo2L) and TNF-*α*  [44, 45].

The death ligand is expressed on the T lymphocytes, natural killer cells, dendritic cells, neutrophils, monocytes or macrophages [46–48]. Membrane-bound TRAIL can be cleaved from the cell surface into a soluble secreted form. Soluble or expressed on immune cells TRAIL plays an important role in surveillance and defense mechanisms against tumor cells [46, 49]. Endogenous TRAIL triggers apoptosis *via *receptor-mediated death (extrinsic pathway) through interaction with the death receptors (DRs) in cancer cells [49–51]. There are two agonistic transmembrane receptors, TRAIL-R1/DR4 and TRAIL-R2/DR5, which bind ligand by extracellular domains. The death receptors contain complete and functional intracellular death domains (DD) responsible for the activation of apoptosis pathway in cancer cells [52, 53].

However, some cancer cells are resistant to TRAIL-induced death [4, 5, 54]. This failure to undergo apoptosis has been implicated in the resistance of cancer cells to TRAIL surveillance and, therefore, in tumor development [49]. The expression of DRs and proapoptotic (Bid, Bax, Bak, Smac/DIABLO) or antiapoptotic (FLIP, Bcl-2, Bcl-xL, Mcl-1, Akt, IAP-1, IAP-2, XIAP, survivin) proteins in cancer cells is involved in TRAIL-resistance [4, 5, 41, 49, 50]. TRAIL-resistant cancer cells can be sensitized to TRAIL-mediated apoptosis by certain polyphenolic compounds [49, 55]. 

## 4. TRAIL Signaling Pathways and the Mechanisms of TRAIL-Resistance in Cancer Cells 

Binding of TRAIL to DRs is the first step of extrinsic apoptotic pathway, also known as the death receptor pathway [56]. The decreased expression of DRs in cancer cell surface causes TRAIL-resistance [57, 58]. Ligation of trimerized TRAIL to TRAIL-R1/DR4 and/or TRAIL-R2/DR5 leads to conformational change in their DD along with the subsequent oligomerization and clustering of the DRs [59, 60]. This DRs activation allows for the recruitment of the adaptor molecule FADD (Fas-associated death domain) with formation of the DISC (death inducing signaling complex), activation of initiator caspases (caspase-8 and -10), cleavage of effector caspases (caspase-3, -6 and -7), and finally DNA fragmentation [61–64]. The antiapoptotic protein FLIP (FLICE (FADD-like IL-1*β*-converting enzyme) inhibitory protein) can also be part of DISC to replace caspase-8 and form an inactive complex. Overexpression of FLIP in cancer cells blocks activation of caspase-8 [4, 5].

**Figure 1:**
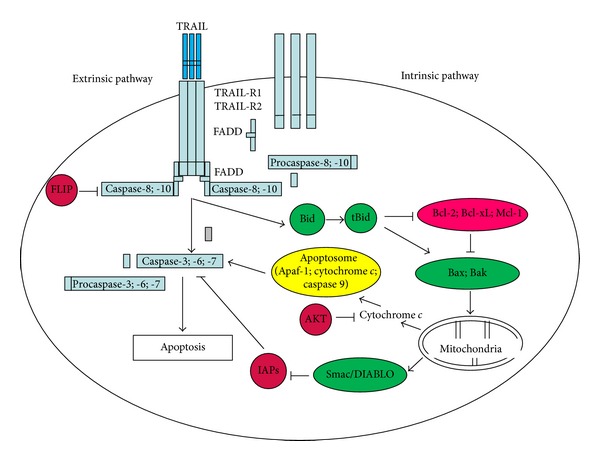
TRAIL-induced apoptotic pathways in cancer cells. TRAIL binds to death receptors, TRAIL-R1 and/or TRAIL-R2 and promotes the recruitment of adaptor molecule FADD (Fas-associated-death domain) to activate caspase-8 and/or caspase-10, which trigger activation of downstream effector caspases (caspase-3, -6, -7). FLIP can block activation of caspase-8 or casapase-10. Caspase-8 mediated also cleavage of Bid (BH3-interacting domain death agonist). Trucated Bid called tBid translocates to the mitochondria where it interacts with proapoptotic Bax and Bak, stimulating disruption of MMP (mitochondrial membrane potential) and the release of cytochrome *c* and Smac/DIABLO (second mitochondrial activator of caspases/direct inhibitor of apoptosis binding protein with low isoelectric point). Antiapoptotic members of Bcl-2 family (Bcl-2, Bcl-xL, and Mcl-1) could inhibit loss of MMP. Akt may prevent cytochrome *c* escape to cytosol. Cytochrome *c* liberated from the mitochondria binds to the adaptor protein Apaf-1 (apoptotic protease-activating factor-1) and procaspase-9, forming the apoptosome and activating caspase-9 which in turn activates executioner caspases (caspase-3, -6, -7) leading to cell death. Activity of executioner caspases is inhibited by IAPs (inhibitor of apoptosis protein): IAP-1, IAP-2, XIAP, and survivin. Smac/DIABLO blocks IAPs.

In some cancer cells activated caspase-8 is sufficient to trigger apoptosis, while other cells require activation of the mitochondrial (intrinsic) pathway to amplify the apoptotic signal. In the mitochondrial pathway, caspase-8 leads indirectly to the activation of effector caspases through the cleavage of the BH3-interacting domain death agonist (Bid), along with the mitochondrial membrane potential (MMP) disruption [49]. Crosstalk between the extrinsic (receptor-dependent) and intrinsic (mitochondrial-dependent) apoptosis pathways is linked by caspase-8-mediated Bid cleavage and subsequent translocation of tBid (truncated Bid) to the mitochondria to initiate the intrinsic apoptosis pathway [1, 2]. Truncated Bid interacts with proapoptotic mitochondrial proteins from Bcl-2 (B-cell leukemia 2) family (Bax, Bak) stimulating the decrease in the MMP [6, 60]. The loss of the integrity of the mitochondrial membrane leads to the release of the cytochrome *c* and the second mitochondrial activator of caspases/direct inhibitor of apoptosis binding protein with low isoelectric point (Smac/DIABLO) [64]. Among the cellular signaling pathways that promote cell survival, antiapoptotic members of Bcl-2 family (Bcl-2, Bcl-xL, Mcl-1) could inhibit the liberation of cytochrome *c* from mitochondria [4, 5]. Akt, a serine/threonine protein kinase, is another important factor contributing TRAIL-resistance. Akt can prevent cytochrome *c* escape to cytosol [47, 48]. Furthermore, cytochrome *c*, in the presence of Apaf-1 (apoptotic protease-activating factor-1) and procaspase-9 forms the apoptosome. Activated caspase-9 stimulates in turn executioner caspases (caspase-3, -6, -7) leading to cell death [10, 49]. Effector caspases activity is controlled by IAPs (inhibitor of apoptosis proteins): IAP-1, IAP-2, XIAP (X-linked inhibitor of apoptosis protein), and survivin. Smac/DIABLO augments apoptosis by binging to the cellular IAP members, which are potent caspase inhibitors [1–5].  Figure 1 demonstrates the TRAIL-induced apoptotic pathways in cancer cells [65].

## 5. Effect of Polyphenolic Components from Propolis on TRAIL-Induced Apoptosis in Cancer Cells

**Figure 2:**
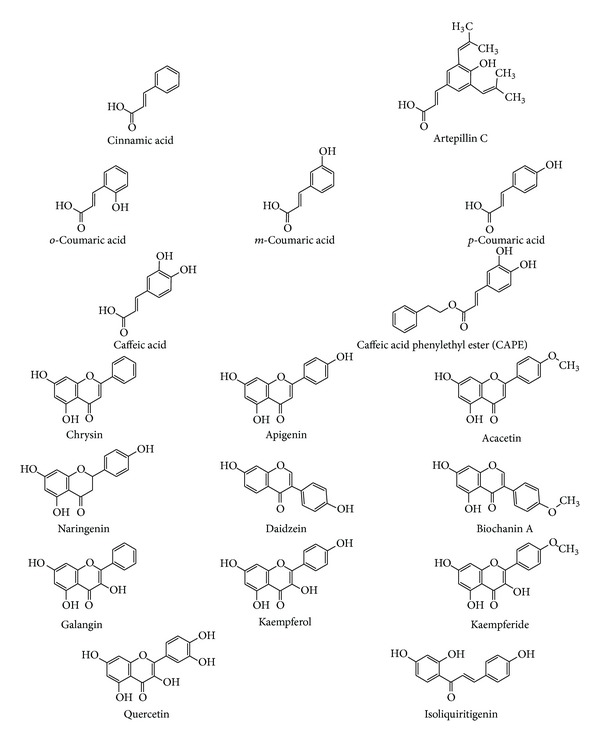
The chemical structures of the main polyphenols from propolis supporting TRAIL-mediated cytotoxicity.

Propolis is a promising raw mixture of natural compounds that should be studied to discover new pharmaceutical products with anticancer and chemopreventive properties [7, 8, 26]. The major active components of propolis are flavonoids and phenolic acids or their esters [9, 13]. Accumulating data clearly indicate that induction of apoptosis is an essential event for chemoprevention of cancer by polyphenolic compounds [36–38]. Inactivation of the TRAIL pathway and escape from the TRAIL-mediated immunosurveillance might play important roles in tumor onset and progression [49]. TRAIL in combination with propolis extracts or with polyphenolic compounds identified in propolis resulted in the synergistic induction of cancer cell death. Our previous findings demonstrated for the first time that ethanolic extracts of European and Brazilian propolis and its polyphenolic constituents overcome TRAIL-resistance in HeLa cervical, LNCaP, and DU145 prostate cancer cells [9–11]. Propolis significantly augments the anticancer activity of TRAIL in cancer cells due to its phenolics. Cinnamic acid and its derivative artepillin C, *o*-coumaric acid, *m*-coumaric acid, *p*-coumaric acid, caffeic acid and its derivative—caffeic acid phenylethyl ester (CAPE), chrysin, apigenin, acacetin, naringenin, daidzein, biochanin A, galangin, kaempferol, kaempferide, quercetin, isoliquiritigenin have been reported to enhance TRAIL-induced death [9–11, 49]. The chemical structures of the components found in propolis supporting TRAIL-mediated cytotoxicity are shown in Figure 2. 

**Figure 3:**
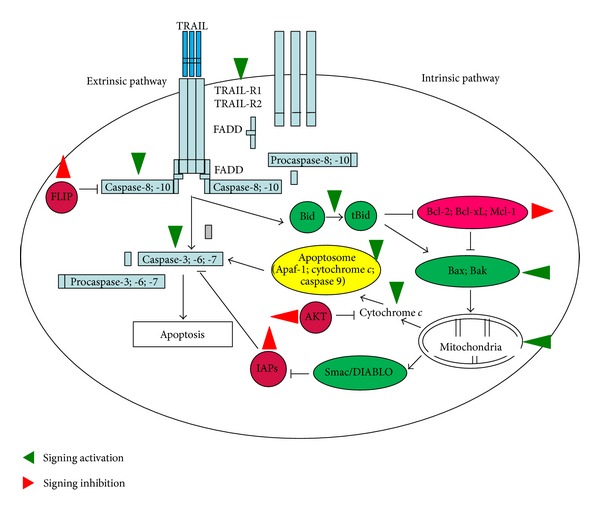
The molecular targets in TRAIL-mediated apoptotic pathways in cancer cells for polyphenols isolated from propolis. Schematic presentation of the mechanisms by which polyphenols detected in propolis modulate TRAIL apoptotic signaling in cancer cell. The green arrows signing activation and red arrows signing inhibition indicate the molecular targets for polyphenols in TRAIL-mediated apoptosis in cancer cells.

TRAIL is a potent inducer of apoptosis in cancer cells. Numerous studies show that many types of cancer cells are resistant to TRAIL-induced death, but combinatorial approaches based on TRAIL and different chemotherapeutic agents, such as small-molecule inhibitors, drugs, and natural compounds, have been developed to overcome the resistance of cancer cells to TRAIL [66, 67]. The decreased expression of death receptors TRAIL-R1 and TRAIL-R2 and antiapoptotic proteins (Bid, Bax, Bak, Smac/DIABLO) or increased expression of antiapoptotic proteins (FLIP, Bcl-2, Bcl-xL, Mcl-1, Akt, IAP-1, IAP-2, XIAP, survivin) in cancer cells were involved in TRAIL-resistance [4, 5]. Recently the molecular mechanism by which the polyphenols identified in propolis sensitize TRAIL-resistant cancer cells are known for artepillin C, chrysin, apigenin, naringenin, daidzein, biochanin A, kaempferol, quercetin, and isoliquiritigenin. The targets in TRAIL-mediated apoptotic pathway for phenolic compounds of propolis are presented in Figure 3. Artepillin C restores TRAIL sensitivity in TRAIL-resistant LNCaP prostate cancer cells by upregulation of TRAIL-R2, activation of caspase-8 and caspase-3, as well as the disruption of MMP [68]. Chrysin and apigenin overcome TRAIL-resistance in MDA-MB-231 breast cancer cells, HT-29 colon cancer cells, HepG2 hepatocellular cancer cells, SK-MEL-37 melanoma cells, Capan-1 pancreatic cancer cells *via* increased expression of TRAIL-R2 and decreased expression of FLIP [69]. In CNE1 nasopharyngeal cancer cells chrysin promotes TRAIL-induced caspase activation (caspase-8 and -3) [70]. Apigenin augments TRAIL-induced apoptosis in Jurkat leukemia T cells, DU145 prostate cancer cells, and DLD-1 colon cancer cells through upregulation of TRAIL-R2, activation of Bid and caspase-8, -10, -9, -3 [71]. Increased expression of TRAIL-R2, induction of Bid cleavage, and loss of MMP in A549 lung cancer cells by naringenin results in significant enhancement of TRAIL-mediated apoptosis [72]. Daidzein reverses TRAIL-resistance in LNCaP prostate cancer stimulating the decrease in the MMP and in LN229 glioma cells activating caspase-9 and downregulating of bcl-2 [73–76]. Biochanin-A sensitizes LNCaP and DU145 prostate cancer cells *via* increased expression of TRAIL-R2 and disruption of MMP [77]. Induction of TRAIL-R1 and TRAIL-R2 expression and caspase-8, -10, -9, -3 activation in SW-480 colon cancer cells and activation of caspase-8, suppression of Akt, survivin, XIAP, and antiapoptotic mitochondrial proteins from Bcl-2 family: Bcl-2, Bcl-xL, Mcl-1 in U251 and U87 glioma cells by kaempferol are sufficient to restore TRAIL sensitivity [78, 79]. Quercetin strongly cooperates with TRAIL to trigger apoptosis in HepG2, SK-Hep, SNU-387, SNU-423, SNU-449, and SNU-475 hepatocellular cancer cells by increased expression of TRAIL-R2 and decreased FLIP expression, in HT-29, SW-620, and Caco-2 colon cancer cells by upregulation of TRAIL-R1 and TRAIL-R2, induction of Bid and caspase-3 cleavage, and release of cytochrome *c* to the cytosol and in U87-MG, U251, A172, and LN229 glioma cells by suppression of survivin, in LNCaP, DU145, and PC3 prostate cancer cells by upregulation of TRAIL-R2, activation of caspase-8, -9 and -3, inhibition of Akt and survivin, in H460, H2009, H1299 and A549 lung cancer cells by increase of TRAIL-R2 expression, activation of caspase-8 and -3, and also by inactivation of Akt and survivin, and in VAL, RL and SUDHL4 B-cell lymphomas cells by down-regulation of survivin and additionally by degradation of Mcl-1, an antiapoptotic mitochondrial protein [80–87]. Isoliquiritigenin upregulates TRAIL-R2 protein levels in cell surface of HT-29 colon cancer and in this way supports TRAIL-mediated apoptosis [88]. Schematic presentations of the mechanisms by which polyphenols from propolis modulate the TRAIL apoptotic signaling in cancer cell are demonstrated in Table 1 and Figure 3.

## 6. Discussion

Epidemiological and preclinical evidence suggests that polyphenols isolated from propolis possess strong cancer chemopreventive activities [7, 8, 26]. It has led to an increased emphasis on cancer prevention strategies in which propolis as the richest source of plant polyphenolics will be used as dietary supplement [10, 11, 49]. In the field of CAM, this paper focuses on the interaction between phenolic components from propolis and TRAIL on tumor cells as the example of immunomodulation through natural substances to be considered for the chemoprevention of neoplasm disease [9–11]. Polyphenols from propolis sensitize tumor cells to TRAIL-induced apoptosis. The compounds exhibit strong cytotoxic effect in combination with TRAIL on cancer cells [65–84]. The TRAIL-mediated apoptotic pathways may be a target of the chemopreventive activity of polyphenols in cancer cells. 

## 7. Conclusion

Targeting TRAIL-induced apoptotic signaling pathway in tumor cells by propolis and its polyphenols is one of the crucial issues in cancer chemoprevention. EEP and its phenolic components sensitize TRAIL-resistant cancer cells and augment anticancer activity of TRAIL. The paper confirms that the overcoming of TRAIL-resistance by propolis and its polyphenols may be one of the mechanisms responsible for their cancer preventive effects. 

## Figures and Tables

**Table 1:** The mechanism by which the polyphenols identified in propolis sensitize TRAIL-resistant cancer cells.

Compounds from propolis	Class of polyphenols	Targets	Cell lines	References
Artepillin C	Cinnamic acid derivative	↑ TRAIL-R2 ↑ caspase-8 ↑ caspase-3	Prostate cancer LNCaP	[68]

Chrysin	Flavone	↑ TRAIL-R2 ↓ FLIP ↑ caspase-8 ↑ caspase-3	Breast cancer MDA-MB-231 Colon cancer HT-29 Hepatocellular cancer HepG2 Melanoma SK-MEL-37 Pancreatic cancer Capan-1 Nasopharyngeal cancer CNE1	[69, 70]

Apigenin	Flavone	↑ TRAIL-R2 ↓ FLIP ↑ Bid cleavage ↑ caspase-8 ↑ caspase-10 ↑ caspase-9 ↑ caspase-3	Breast cancer MDA-MB-231 Colon cancer HT-29 Hepatocellular cancer HepG2 Melanoma SK-MEL-37 Pancreatic cancer Capan-1 Leukemia Jurkat Prostate cancer DU145 Colon cancer DLD-1	[69, 71]

Naringenin	Flavanone	↑ TRAIL-R2 ↑ Bid cleavage ↑ loss of MMP	Lung cancer A549	[72]

Daidzein	Isoflavone	↑ loss of MMP ↑ caspase-9 ↓ Bcl-2	Prostate cancer LNCaP Glioma LN229	[73–76]

Biochanin A	Isoflavone	↑ TRAIL-R2 ↑ loss of MMP	Prostate cancer LNCaP Prostate cancer DU145	[77]

Kaempferol	Flavanol	↑ TRAIL-R1 ↑ TRAIL-R2 ↑ caspase-8 ↑ caspase-10 ↑ caspase-9 ↑ caspase-3 ↓ Akt ↓ survivin ↓ XIAP ↓ Bcl-2 ↓ Bcl-xL ↓ Mcl-1	Colon cancer SW-480 Glioma U251 Glioma U87	[78, 79]

Quercetin	Flavanol	↑ TRAIL-R1 ↑ TRAIL-R2 ↓ FLIP ↑ Bid cleavage ↓ Mcl-1 ↑ cytochrome *c* release ↓ Akt ↓ survivin ↑ caspase-8 ↑ caspase-9 ↑ caspase-3	Hepatocellular cancer HepG2 Hepatocellular cancer SK-Hep Hepatocellular cancer SNU-387 Hepatocellular cancer SNU-423 Hepatocellular cancer SNU-449 Hepatocellular cancer SNU-475 Colon cancer HT-29 Colon cancer SW-620 Colon cancer Caco-2 Glioma U87-MG Glioma U251 Glioma A172 Glioma LN229 Prostate cancer LNCaP Prostate cancer DU145 Prostate cancer PC3 Lung cancer H460 Lung cancer H2009 Lung cancer H1299 Lung cancer A549	[80–87]
			Lymphoma B VAL Lymphoma B RL Lymphoma B SUDHL4	

Isoliquiritigenin	Chalcone	↑ TRAIL-R2	Colon cancer HT-29	[88]
